# Role of human RNase 7 in neuronal and glial cell models: moving towards an unexpected new functional link

**DOI:** 10.1111/febs.70484

**Published:** 2026-03-11

**Authors:** Rosanna Culurciello, Maria Cristiano, Ilaria Di Nardo, Ida Palumbo, Erika Piccolo, Rosario Oliva, Andrea Bosso, Aldo Donizetti, Eugenio Notomista, Elio Pizzo

**Affiliations:** ^1^ Department of Biology University of Naples Federico II Italy; ^2^ Department of Chemical Sciences University of Naples Federico II Italy; ^3^ Center of Advanced Measurement and Technology Services (CeSMA) Federico II University Naples Italy

**Keywords:** immunomodulation, infections, LPS, neuronal cells, RNase 7

## Abstract

Human ribonuclease 7 (RNase 7), originally isolated from skin, is a member of the RNase A Superfamily and primarily known to be among the endogenous proteins with the most pronounced antimicrobial properties. Nevertheless, to date, studies on this enzyme are mostly limited to its antimicrobial effects on epithelial tissue, which is surprising considering the numerous districts of the human body potentially susceptible to infections. This inference inspired the present work, which is mainly dedicated to uncovering new roles of the RNase 7 in human cells that have not yet been explored in relation to this antimicrobial agent: neuronal cells. In this context, we decided to address possible host defense properties of RNase 7 in the nervous system, and to do this, we have selected both neuroblastoma SH‐SY5Y and glioblastoma U‐87 MG cells as experimental models. As a result, we found that, in addition to endogenously expressing RNase 7 under altered growth conditions, both cell lines are also responsive to its administration. More specifically, we highlighted for the first time that recombinant RNase 7 reduces the expression levels of pro‐inflammatory cytokines, the release of nitric oxide, and the production of reactive oxygen species in LPS‐stimulated SH‐SY5Y and U‐87 MG cells, contributing to their innate immune response. Moreover, here we highlighted that internalized recombinant RNase 7 can contribute to bacterial clearance in both SH‐SY5Y and U‐87 MG. These findings extend the functional role of RNase 7 beyond epithelia, indicating its potential involvement in neuroimmune regulation and suggesting novel therapeutic implications.

## Graphical abstract

Human RNase 7 is known to exert antimicrobial activity in epithelial tissues. Here, using SH‐SY5Y and U‐87 MG, neuroblastoma and glioblastoma cell lines, respectively, we found that RNase 7 enhances immune responses to LPS stimulation by reducing the expression of pro‐inflammatory cytokines, nitric oxide, and ROS. It also promotes bacterial clearance. These findings suggest RNase 7 has neuroimmune functions, pointing to its potential therapeutic role in neuroinflammatory conditions.

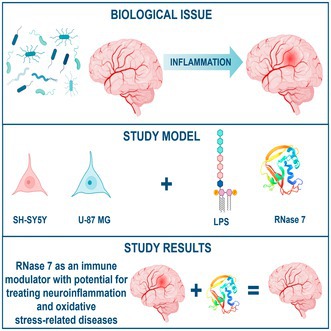

**Abbreviations**
BSA: bovine serum albumin
CFU: colony‐forming unit
CLSM: confocal laser scanning microscopy
CNS: central nervous system
DAMPs: damage‐associated molecular patterns
DAPI: 4′,6‐diamidino‐2‐phenylindole, dihydrochloride
DCFH‐Da: 2′,7′‐dichlorofluorescin diacetate
DMEM: Dulbecco's modified Eagle medium
ECL: enhanced chemiluminescence substrate
ECP: eosinophil cationic protein
EDN: eosinophil‐derived neurotoxin
ELISA: enzyme‐linked immunosorbent assays
FBS: fetal bovine serum
GAPDH: glyceraldehyde 3‐phosphate dehydrogenase
H_2_O_2_
: hydrogen peroxide
HPLC: high‐performance liquid chromatography
HRP: horseradish peroxidase
HSV: herpes simplex virus
IBs: inclusion bodies
IgG: immunoglobulin G
IL‐1: interleukin‐1
IL‐1β: interleukin‐1β
IL‐6: interleukin‐6
IPTG: isopropyl β‐D‐1‐thiogalactopyranoside
Kd: dissociation constant
LB: Luria‐Bertani broth
LPS: lipopolysaccharide
MBC: minimum bactericidal concentration
MDA‐equivalents: malondialdehyde‐equivalents
MIC: minimum inhibitory concentration
MOI: multiplicity of infection
mRNAs: messenger RNAs
MTT: 3‐(4,5‐dimethyl‐2‐thiazolyl)‐2,5‐diphenyl‐2H‐tetrazolium bromide
NB: nutrient broth
NO: nitric oxide
PABP: poly‐A binding protein
PAMPs: pathogen‐associated molecular patterns
PBS: phosphate‐buffered saline
PFA: paraformaldehyde
PVDF: polyvinylidene fluoride
RA: retinoic acid
RI: ribonuclease inhibitor
RNA: ribonucleic acid
RNase: ribonuclease
ROS: reactive oxygen species
RT‐qPCR: reverse transcription quantitative polymerase chain reaction
SA: sodium arsenite
SDS/PAGE: sodium dodecyl sulfate‐polyacrylamide gel electrophoresis
TBARS: thiobarbituric acid reactive substances
TCA: trichloroacetic acid
TLR4: toll‐like receptor 4
TMB: 3,3′,5,5′‐tetramethylbenzidine
TNF‐α: tumor necrosis factor‐α

## Introduction

The human body hosts an extraordinarily complex community of commensal bacteria, and increasing evidence corroborates the idea that microbes residing in the various districts actively participate in shaping and maintaining the correct physiology during development and homeostasis, basically functioning as an accessory “organ” [1, 2]. In contrast, pathogenic bacteria continually refine molecular strategies to survive within hosts, damaging physiological function and fitness through secreted toxins and metabolites [3, 4].

Among the host‐derived factors that counteract microbial threats and can possibly contribute to the fine‐tuned balance between symbiosis and infection, different members of the Ribonuclease A (RNase A) Superfamily have emerged as key players [5].

RNase A Superfamily includes an extensive group of vertebrate‐specific proteins that share a stable three‐dimensional disulfide‐bonded structure and the ability to catalyze the degradation of RNA by means of a catalytic triad composed of two histidines and one lysine [6, 7]. Beyond that, all these stable enzymes can serve a variety of diverse biological roles *in vivo*, not necessarily linked to catalytic properties, including host defense, neurotoxicity, promotion of proliferation, anti‐apoptosis, and regulation of cellular homeostasis [8]. The biological activities of many of these enzymes, including RNase 7, are significantly enhanced by their nature as secreted proteins. This secretory pathway is mediated by an N‐terminal signal peptide [9] and once secreted, these enzymes exert their functions as paracrine factors, influencing the behavior of adjacent cells and contributing to localized immune modulation and tissue homeostasis [10].

Host defense functions in mammalian members of the Superfamily are mainly attributed to eosinophil secretory ribonucleases EDN and ECP [11, 12], RNase 6 [13, 14] and RNase 7, the latter a bactericidal cationic protein initially isolated from human skin [15].

To date, the exact molecular mechanism underlying the antimicrobial activity of RNase 7 is not fully understood. It is clearly reported that its RNA‐degrading activity is not essential for its bactericidal effect, while cationic residues seem to play a decisive role [15, 16, 17]. Indeed, various functional studies suggest that the antimicrobial properties of RNase 7 are mainly associated with cationic residues in its N‐terminal domain [18], a feature common to many antimicrobial active members of RNase A superfamily [19, 20]. Accordingly, the proposed mechanism of action of RNase 7 involves its binding to the negatively charged bacterial surface via electrostatic interactions followed by membrane disruption [16, 19]. Supporting this hypothesis, RNase 7 has been shown to depolarize the cytoplasmic membrane of *Escherichia coli* and *Staphylococcus aureus* and to interact with LPS and peptidoglycan [21, 22], major components of the cell wall of Gram‐negative and Gram‐positive bacteria, respectively [23].

Thanks to its antimicrobial properties, RNase 7 plays a significant role in the innate immune response [24], to date, mainly observed in the skin, urinary [19], and respiratory tract [20, 25]. In addition to the direct cytotoxic action against bacteria, it exhibits a wide range of immunomodulatory functions, including (a) inflammatory response, by influencing cytokine production and immune cell recruitment; (b) cell signaling, interacting with immune cells, potentially affecting their activation and function; (c) barrier function in epithelial tissues, where RNase 7 contributes to maintaining the integrity of barrier functions, which is crucial for preventing pathogen entry [6, 15, 26, 27].

Overall, RNase 7 plays a key role in host defense [28] and for this reason, its multiple properties make it a valuable target for research in immunology and therapeutic development.

Although RNase 7 is expressed in many tissues of the human body [25], it is rather well characterized for most of the functions described above, mainly in the skin [29] and lining epithelia, in which many authors have focused on deepening its functions in the immune system and response to pathogens [30, 31].

This inspired the current study, which investigates the functional relationship between RNase 7 and human cells, particularly neurons and glial cells, both vulnerable to microbial infections but also unexplored to date in relation to this enzyme.

Neurons, the key units of the brain and nervous system, process sensory input, drive motor output, and relay electrical signals throughout. Treating infections in this system remains a challenge, largely due to the blood–brain barrier, which restricts access to many standard antibiotics. These infections, often marked by inflammation and oxidative stress, demand therapeutics that not only target pathogens but also modulate neuroinflammation and penetrate neural tissue with precision.

There is little evidence that RNase 7 may play a potential role in the nervous system as well, despite it being a vulnerable target for several infections, such as meningitis, encephalitis, myelitis, brain abscesses, and neurosyphilis, which can affect the brain and/or the spinal cord. Nervous system infections can be very serious and, in some cases, life‐threatening, so proper treatment can make a big difference in outcomes.

The interaction between the nervous system and inflammatory processes is a rapidly growing field of research, highlighting how inflammation can influence neurological function and *vice versa*. Both acute and chronic inflammation are involved in many neurodegenerative diseases [32].

Neuroinflammation, also known as central nervous system (CNS) inflammation, is an immune response triggered by various harmful stimuli, including infection, trauma, ischemia, neurodegenerative diseases, and metabolic dysfunction. The CNS, which includes the brain and spinal cord, has a unique immune system mainly regulated by glial cells, astrocytes, and microglia, all essential for maintaining neuronal homeostasis and performing immune defense functions.

When the CNS is subjected to an insult, microglial cells are activated and release a variety of inflammatory mediators, including pro‐inflammatory cytokines like TNF‐α (Tumor necrosis factor‐α), IL‐1β (Interleukin 1β), and IL‐6 (Interleukin 6), adhesion molecules, and ROS (Reactive Oxygen Species). This initial response is crucial to protect the brain from acute injury; however, chronic or dysfunctional inflammation can lead to permanent damage to nervous tissue [33].

Molecules with antimicrobial properties could play a protective role in neuroinflammation. Examples of this include proteins such as lysozyme and defensins, which have been shown to possess anti‐inflammatory properties against pathogens in the CNS [34, 35].

The initial response to infection involves immune cells, including macrophages and dendritic cells, detecting pathogens or tissue damage via specialized receptors that recognize PAMPs and DAMPs. Then damaged cells release cytokines and chemokines, activating other immune cells and recruiting new arrivals to the site of inflammation. Pro‐inflammatory cytokines like TNF‐α or IL‐1 (Interleukin 1) cause blood vessels to dilate, increasing blood flow to the affected area.

The same cytokines increase vascular permeability, allowing the passage of cells and plasma proteins into the damaged tissue. Neutrophils and monocytes migrate into the inflamed area to phagocytose pathogens and cellular debris, a process mediated by chemotaxis.

Activated immune cells engulf and degrade pathogens and cellular debris through the process of phagocytosis. These cells also produce cytokines, prostaglandins, and leukotrienes, which amplify and modulate the inflammatory response.

After elimination of the harmful agent, fibroblasts and astrocytes promote tissue repair and the formation of new extracellular matrix. If the infection persists, the adaptive immune system is activated, involving T and B lymphocytes, with the production of specific antibodies [36, 37, 38].

RNase 7 may be theoretically involved in nervous system defense processes against viral and bacterial infections, helping to maintain the integrity and health of neural tissue. Additionally, various studies indicate a possible link between RNase 7 and inflammatory processes [27, 29, 39, 40] corroborating with these possible implications, also in neurodegenerative conditions or inflammatory diseases of the nervous system.

Due to the current lack of knowledge on the potential role of RNase 7 in the nervous system, this pilot study focused on exploring a possible functional role for the enzyme in the brain using two cell lines: SH‐SY5Y neuroblastoma and U‐87 MG glioblastoma. We confirmed that both undifferentiated and differentiated SH‐SY5Y cells, as well as U‐87 MG cells, endogenously express RNase 7 both in physiological conditions and following different stimuli. Furthermore, our findings highlight that the recombinant protein can exert potent immunomodulatory effects on lipopolysaccharide (LPS)‐stimulated nervous cells and enhance intracellular bacterial clearance. These pioneering data introduce a new research front for RNase 7, suggesting a potential therapeutic role in the defense of the nervous system from microbial infections.

## Results

### Expression analysis of endogenous RNase 7 in SH‐SY5Y and U‐87 MG cells

The first test to verify a potential connection between RNase 7 and the nervous system was based on detecting the presence of the protein in SH‐SY5Y and U‐87 MG cells.

**Fig. 1:**
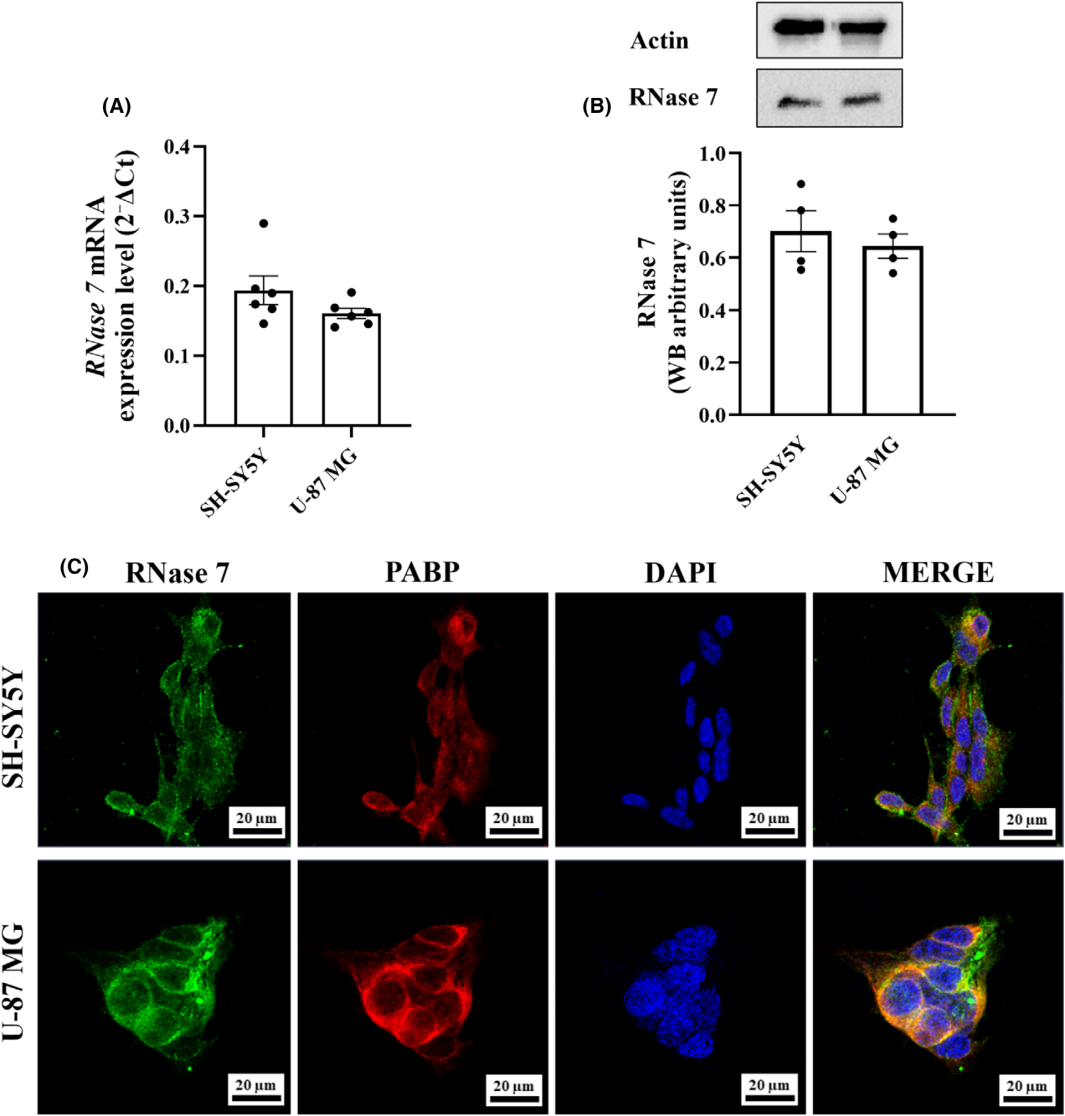
Expression analysis of RNase 7 in SH‐SY5Y and U‐87 MG cells. (A) RT‐qPCR of mRNA encoding RNase 7. The experiments were performed in triplicate, each on *n* = 2 biological replicates, and reported as mean ± SEM; (B) Western blot analysis of protein extracts from SH‐SY5Y and U‐87 MG cells. The experiments were performed in duplicate, each on *n* = 2 biological replicates, and reported as mean ± SEM. (C) Immunofluorescence analysis of SH‐SY5Y and U‐87 MG cells (green: RNase 7; red: PABP; blue: DAPI nuclear staining). Statistical analyses were carried out by one‐way ANOVA, followed by Bonferroni's post‐test. See Materials and methods section for experimental details.

As shown in Fig. 1 (Panels A and B), analyses performed by RT‐qPCR and western blotting clearly indicate, to our knowledge for the first time, that endogenous *RNase 7* is expressed in SH‐SY5Y and U‐87 MG cells. Furthermore, immunofluorescence analyses have highlighted a purely cytosolic localization of the protein in both cell lines (Fig. 1, Panel C). Altogether, these findings support the idea that this ribonuclease can play possible functional roles in this previously unexplored cellular context.

Since RNase 7 expression and secretion are known to be regulated in various conditions, such as infection, inflammation, and oxidative stress in other tissues [20], it seemed essential to verify whether the protein exhibited the same behavior also in the cell types under investigation.

**Fig. 2:**
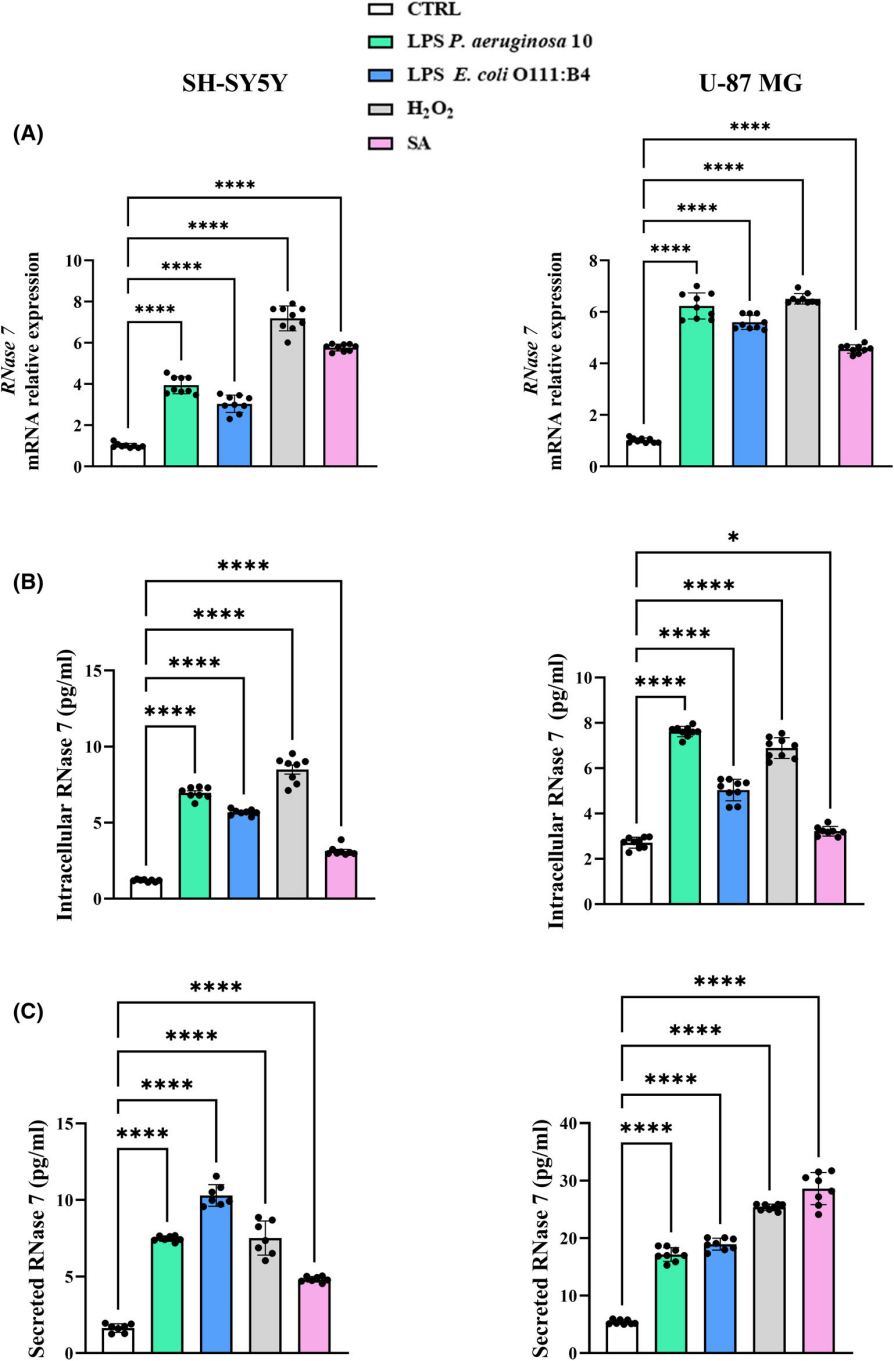
Analysis of RNase 7 expression level in SH‐SY5Y and U‐87 MG cells cultured under normal or altered growth conditions. Data show that RNase 7 is upregulated and secreted by SH‐SY5Y and U‐87 MG cells in response to both pro‐inflammatory and oxidative stimuli. (A) RT‐qPCR analysis of mRNA encoding RNase 7. The experiments were performed in triplicate each on *n* = 3 biological replicates, and reported as mean ± SEM; (B) ELISA assay on cellular protein extracts. The experiments were performed in quadrupled each on *n* = 2 biological replicates for SH‐SY5Y cells and in triplicate each on *n* = 3 biological replicates for U87‐MG cells and reported as mean ± SEM; (C) ELISA assay on proteins from conditioned culture medium. The experiments were performed in quadrupled each on *n* = 2 biological replicates and reported as mean ± SEM. Any evident outlier has been discarded. CTRL: unstimulated control cells; LPS from *Pseudomonas aeruginosa* 10 and LPS from *Escherichia coli* O111:B4 were both administered at a dose equal to 1 μg·mL^−1^ for 24 h; H_2_O_2_ was administered at a dose equal to 100 μm for 24 h; SA (sodium arsenite) was administered at a dose equal to 500 μm for 1 h. The experiments were performed in triplicate, each on *n* = 3 biological replicates, and reported as mean ± SEM. Statistical analyses were carried out by one‐way ANOVA, followed by Bonferroni's post‐test (*P < 0.05 or ****P < 0.0001). See Materials and methods section for experimental details.

As shown in Fig. 2, both in SH‐SY5Y and U‐87 MG cells exposed to altered growth conditions, such as inflammatory triggered by LPS and pro‐oxidative stimuli induced by H_2_O_2_ or sodium arsenite, *RNase 7* gene expression (Panel A), protein synthesis (Panel B), and its secretion (Panel C) were all significantly upregulated.

**Fig. 3:**
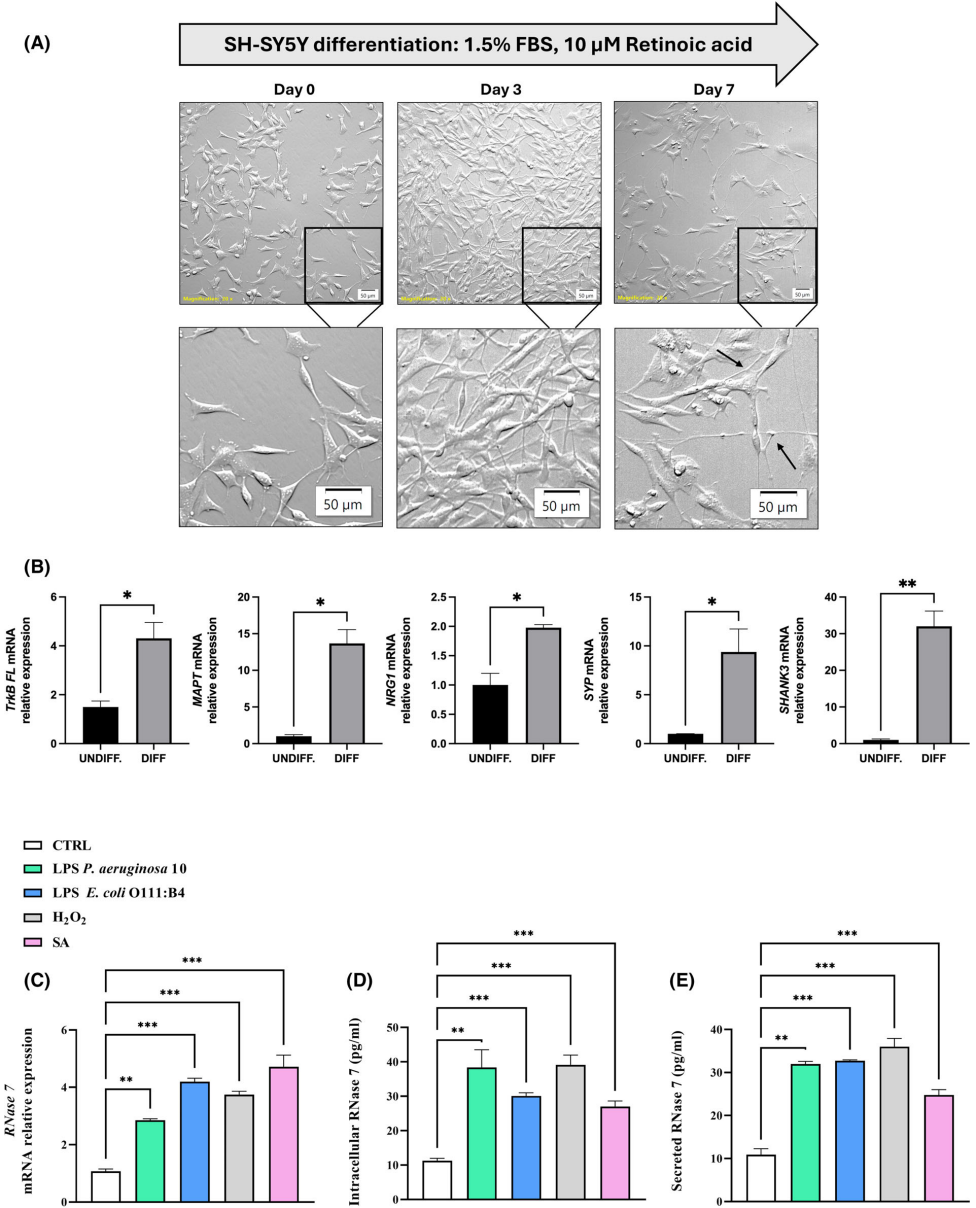
Analysis of morphology (A), expression of neuronal differentiation markers (B) and RNase 7 (C–E) in SH‐SY5Y cells subjected to 7‐day treatment with 1.5% FBS and 10 μm RA. (A) Bright‐field images of SH‐SY5Y cells acquired during their differentiation. Black arrows indicate neurites; (B) Gene expression analysis by RT‐qPCR of neuronal differentiation markers in SH‐SY5Y cells. Undiff: undifferentiated cells; Diff: differentiated cells; (C) RT‐qPCR analysis of mRNA encoding RNase7; (D) ELISA assay on cellular protein extracts; (E) ELISA assay on proteins extracted from conditioned culture medium. CTRL: unstimulated cells control; LPS from *Pseudomonas aeruginosa* 10 and LPS from *Escherichia coli* O111:B4 were administered at a dose equal to 1 μg·mL^−1^ for 24 h; H_2_O_2_ was administered at a dose equal to 100 μm for 24 h; SA was administered at a dose equal to 500 μm for 1 h. The experiments were performed in triplicate and reported as mean ± SEM. Statistical analyses were carried out by one‐way ANOVA, followed by Bonferroni's post‐test (*P < 0.05, **P < 0.01 or ***P < 0.001). See Materials and methods section for experimental details.

To further corroborate this behavior, the same experiment has been conducted on retinoic acid (RA)‐differentiated SH‐SY5Y cells. To this aim, cells were treated with 1.5% FBS and 10 μm of retinoic acid (RA) for 7 days to induce an appropriate cell differentiation. In Fig. 3 (Panels A and B) it is possible to observe both morphological changes of SH‐SY5Y and the expression of neuronal differentiation markers, respectively. It was also observed that in differentiated SH‐SY5Y cells the *RNase 7* gene expression levels, protein production, and secretion (Fig. 3, Panels C–E) were comparable with those observed in undifferentiated cells (Fig. 2).

### Immunomodulatory properties of recombinant RNase 7

LPS, specific endotoxins of Gram‐negative strains, are potent activators of the innate immune response, primarily through their binding to Toll‐like receptor 4 (TLR4), which triggers downstream signaling cascades and promotes the production of pro‐inflammatory cytokines, also within the nervous system.

Once it was established that RNase 7 presented an expression and secretion profile in neurons and astrocytes subjected to altered growth conditions, including treatment with LPS, we proceeded to analyze the potential immunomodulatory properties of the enzyme on LPS‐stimulated SH‐SY5Y and U‐87 MG cells. To this aim, a recombinant version of RNase 7 was produced in *E. coli* (see Materials and methods section). The infection procedures we established involved for both cell lines the use of 2 different types of LPS, respectively isolated from *Pseudomonas aeruginosa* 10 and *E. coli* O111:B4, and used at a dose equal to 1 μg·mL^−1^. Based on this setup, to investigate the potential anti‐inflammatory properties of RNase 7, we measured (see Materials and methods) the release of a series of inflammatory mediators, including nitric oxide (NO), reactive oxygen species (ROS), and cytokines such as TNF‐α and IL‐6 in SH‐SY5Y and U‐87 MG cells subjected to co‐treatment with LPS and RNase 7.

**Fig. 4:**
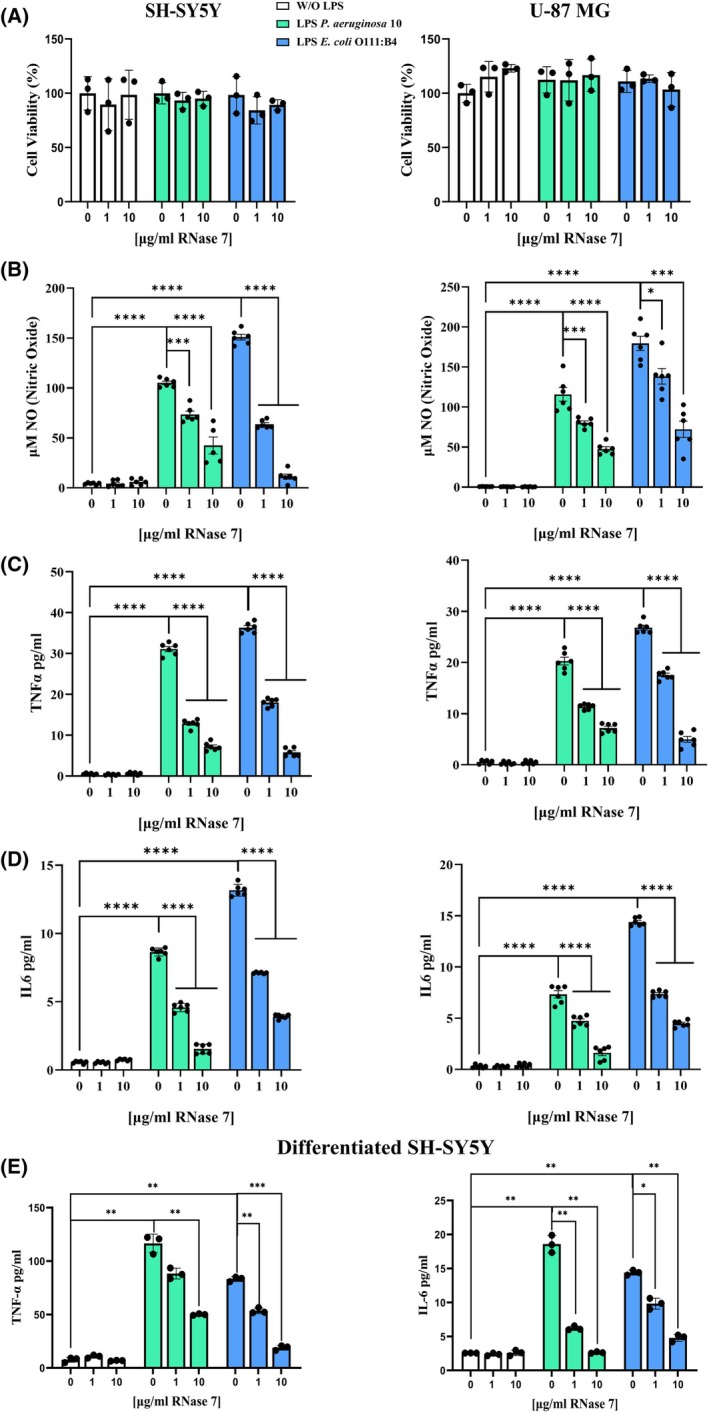
Anti‐inflammatory potential of RNase 7 in SH‐SY5Y, U‐87 MG and differentiated SH‐SY5Y cells stimulated with LPS from *Pseudomonas aeruginosa* 10 and LPS from *Escherichia coli* O111:B4. Data show that recombinant RNase 7 does not exert any toxic effects and suppresses the release of key inflammatory mediators induced by bacterial LPS. (A) Non‐toxicity of recombinant RNase 7 and LPS in SH‐SY5Y and U‐87 MG under experimental conditions used in immunomodulation assays. Cell viability was determined by MTT assay. The experiments were performed in monoplicate each on *n* = 3 biological replicates and reported as mean ± SEM. (B) Analysis of NO release by Griess assay. The experiments were performed in triplicate, each on *n* = 2 biological replicates, and reported as mean ± SEM. Any evident outlier has been discarded. (C) ELISA assay for the evaluation of TNF‐α release. The experiments were performed in triplicate, each on *n* = 2 biological replicates, and reported as mean ± SEM. (D) ELISA assay for the evaluation of IL‐6 release. The experiments were performed in triplicate, each on *n* = 2 biological replicates, and reported as mean ± SEM. (E) ELISA assay for the evaluation of TNF‐α release (on the left) and of IL‐6 release (on the right) in differentiated SH‐SY5Y cells stimulated with LPS and RNase 7. The experiments were performed in monoplicate each on *n* = 3 biological replicates, and reported as mean ± SEM. W/O LPS: unstimulated cells control; LPS from *P. aeruginosa* 10 and LPS from *E. coli* O111:B4 were administered at a dose equal to 1 μg·mL^−1^ for 24 h; RNase 7 (1 and 10 μg·mL^−1^ for 24 h) were tested alone or in presence of LPS. Statistical analyses were carried out by one‐way ANOVA, followed by Bonferroni's post‐test (*P < 0.05, **P < 0.01, ***P < 0.001 or ****P < 0.0001). See Materials and methods section for experimental details. Any evident outlier has been discarded.

SH‐SY5Y and U‐87 MG cells were stimulated with a mixture composed of 1 μg·mL^−1^ LPS from *P. aeruginosa* 10 and RNAse 7 (1 or 10 μg·mL^−1^) or of 1 μg·mL^−1^ LPS from *E. coli* O111:B4 and RNAse 7 (1 or 10 μg·mL^−1^) for 24 h. The collected results clearly highlighted that without exerting any toxic effect (Fig. 4 Panel A), RNase 7 alone did not induce the release of pro‐inflammatory factors. However, when co‐administered with LPS, RNase 7 was able to effectively inhibit in a dose‐dependent manner the release of NO, TNF‐α, and IL‐6 (Fig. 4 Panels B, C and D). Interestingly, similar trends were observed also in RA‐differentiated SH‐SY5Y cells (see Fig. 4 Panel E). Overall, the observed results strongly suggest that RNase 7 may act as an LPS inhibitor.

**Fig. 5:**
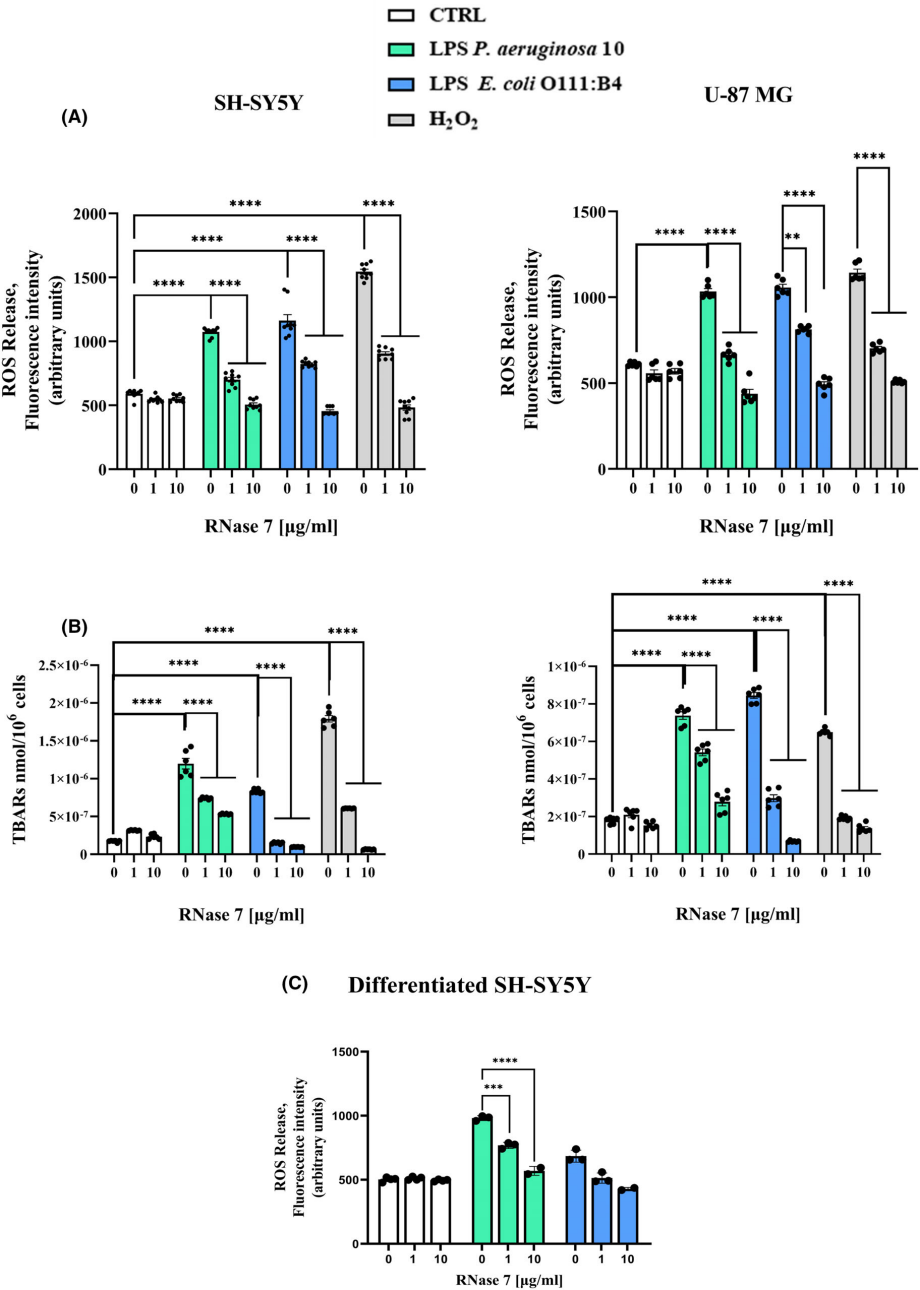
Antioxidant activity analysis of RNase 7 on SH‐SY5Y, U‐87 MG and differentiated SH‐SY5Y cells. Data indicate that RNase 7 counteracts oxidative damage induced by inflammatory and oxidative stimuli. (A) ROS release detection by DCFH‐Da assay. The experiments were performed in triplicate each on *n* = 3 biological replicates for SH‐SY5Y cells and in triplicate each on *n* = 2 biological replicates for U87‐MG cells and reported as mean ± SEM; (B) Evaluation of ROS‐induced lipid peroxidation by TBARS assay. The experiments were performed in triplicate, each on *n* = 2 biological replicates, and reported as mean ± SEM. (C) ROS release analyzed by DCFH‐Da assay on differentiated SH‐SY5Y The experiments were performed in monoplicate each on *n* = 4 biological replicates, and reported as mean ± SEM. Any evident outlier has been discarded (CTRL: unstimulated control cells; LPS from *Pseudomonas aeruginosa* 10 and LPS from *Escherichia coli* O111:B4 were administered at a dose equal to 1 μg·mL^−1^ for 24 h; H_2_O_2_ was assayed at a dose equal to 100 μm for 24 h). Statistical analyses were carried out by one‐way ANOVA, followed by Bonferroni's post‐test (**P < 0.01, ***P < 0.001 or ****P < 0.0001). See Materials and methods section for experimental details.

Moreover, when RNase 7 was tested for its possible role in balancing redox homeostasis in LPS‐stimulated SH‐SY5Y and U‐87 MG cells, we found that it was able to modulate both the ROS production and their secondary damages on lipid peroxidation (Fig. 5, Panels A and B); interestingly, we detected that RNase 7 was also able to limit ROS release in cells directly exposed to a strong oxidant agent as H_2_O_2_ (Fig. 5, Panels A and B, gray histograms). To confirm whether differentiated cells behaved similarly, ROS levels were also measured in RA‐differentiated SH‐SY5Y cells stimulated with LPS (Fig. 5 Panel C), showing a trend consistent with that observed in undifferentiated cells. These results clearly indicate that RNase 7 can act as a modulator of immune responses induced by different stimuli, demonstrating an attitude already shown by other stress‐induced RNases belonging to the RNase A Superfamily [41, 42].

### Evaluation of RNase 7 binding to LPSs

**Fig. 6:**
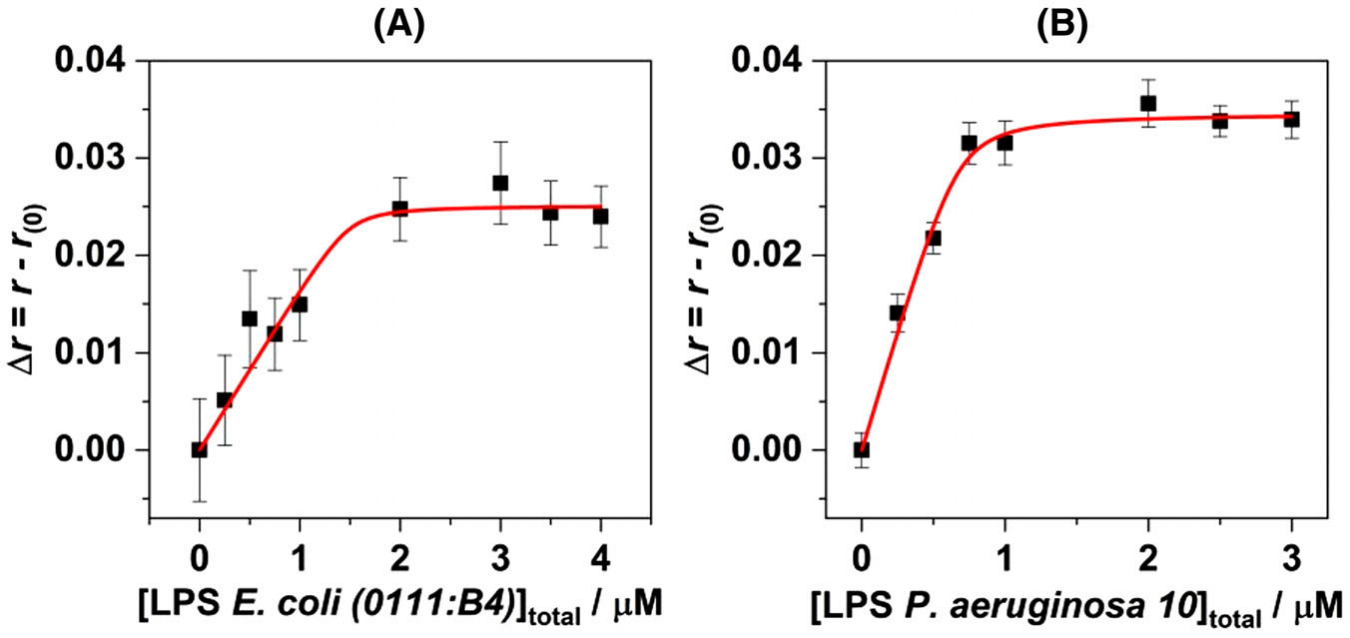
LPS binding analysis. Binding isotherms obtained by means of steady‐state fluorescence anisotropy titration experiments of RNase 7 with (A) LPS from *Escherichia coli* O111:B4 and (B) LPS from *Pseudomonas aeruginosa* 10. The excitation wavelength was 280 nm, and the anisotropy of RNase 7 was evaluated at 338 nm. The solid red lines represent the best fit of experimental data according to an equivalent and independent sites model equation. All the experiments were performed at a temperature of 25 °C in 10 mm sodium phosphate buffer, pH 7.4. The experiments were performed on *n* = 10 replicates, and reported as mean ± SD.

As the observed results on LPS‐stimulated SH‐SY5Y and U‐87 MG cells (Figs 4 and 5) would suggest that RNase 7 can act as an LPS scavenger, fluorescence anisotropy experiments were performed to verify a possible interaction between the enzyme and LPS in the monomeric (non‐micellar) form and to evaluate it quantitatively (*i.e*., determining the dissociation constant, *K*_d_). In Fig. 6, the binding isotherms obtained from titration experiments of RNase 7 with LPS *E. coli* O111:B4 (Panel A) and with LPS *P. aeruginosa* 10 (Panel B) are reported.

Data analysis revealed that the values of the *K*_d_ were 14.1 ± 7.0 nm and 24.2 ± 10.0 nm for the formation of a complex between RNase 7 and LPS from *E. coli* O111:B4 or LPS from *P. aeruginosa* 10, respectively. Of note, our analysis suggests that the protein can form a 1 : 1 complex with LPS from *E. coli* O111:B4. Instead, the complex of RNase 7 with LPS *P. aeruginosa* 10 is 2 : 1. It is known that the LPS structure is unique for each bacterium [43]. Thus, these differences could be attributed to the different structures adopted by the two LPS. Collectively, our binding experiments suggest the presence of a very strong interaction of the protein with both LPS in the monomeric state.

**Fig. 7:**
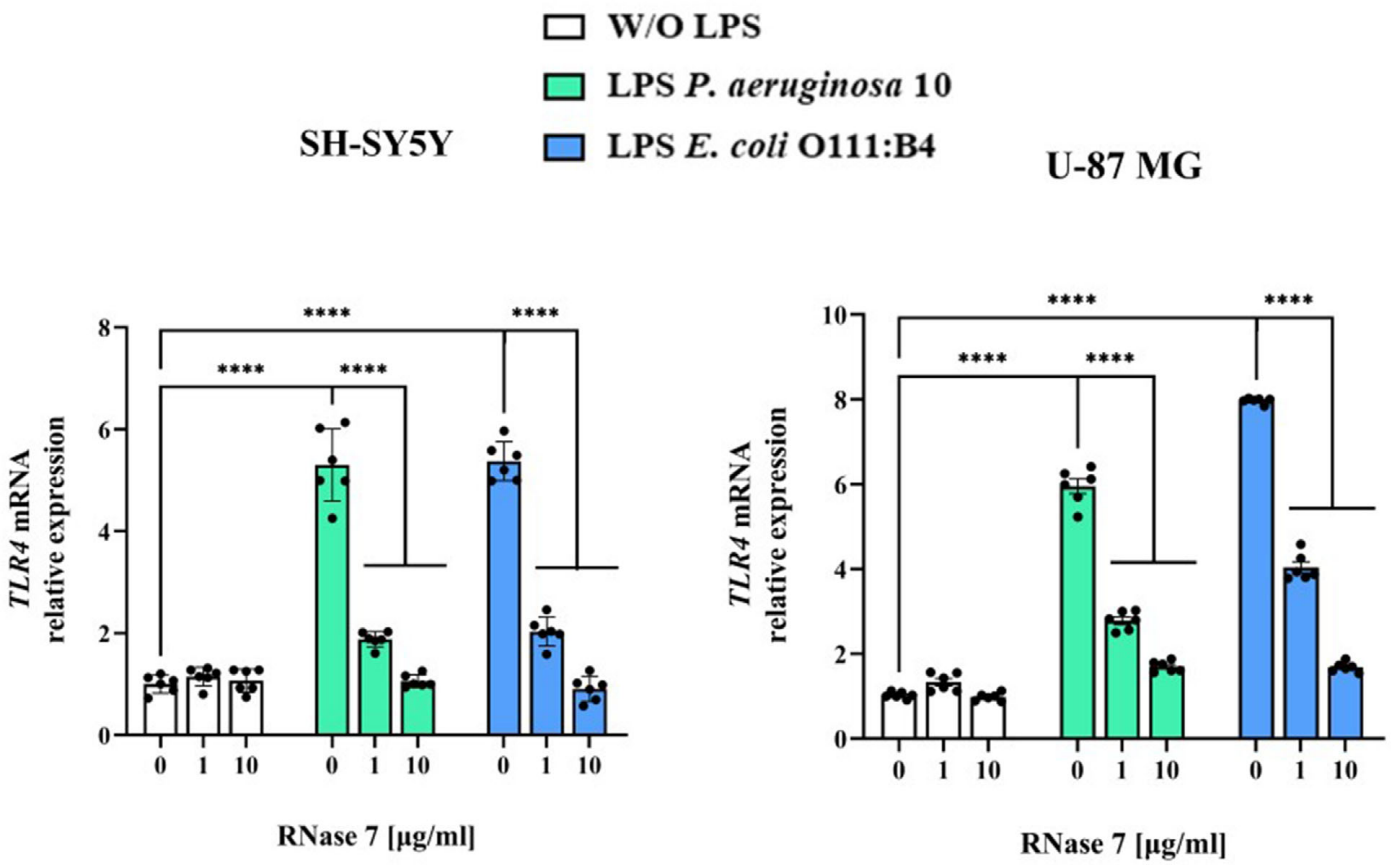
Analysis by RT‐qPCR of TLR4 expression level in SH‐SY5Y and U‐87 MG cells cultured in different altered growth conditions. RNase 7 downregulates TLR4 gene expression in LPS‐treated cells, indicating a potential immunomodulatory role in the cellular response to endotoxin. (W/O LPS: unstimulated control cells; LPS from *Pseudomonas aeruginosa* 10 and LPS from *Escherichia coli* O111:B4 were administered at a dose equal to 1 μg·mL^−1^ for 24 h). The experiments were performed in triplicate, each on *n* = 2 biological replicates, and reported as mean ± SEM. Statistical analyses were carried out by one‐way ANOVA, followed by Bonferroni's post‐test (****P < 0.0001). See Materials and methods section for experimental details.

The ability of RNase 7 to interact with LPS is further underlined by the markedly reduced expression of the *TLR4* gene observed in both SH‐SY5Y and U‐87 MG cells, compared to cells exposed to LPS alone (Fig. 7). This suggests that RNase 7 may sequester or neutralize LPS before it can effectively engage TLR4, thereby dampening the receptor's downstream signaling cascade [44]. Given the pivotal role of TLR4 in detecting bacterial endotoxins and triggering pro‐inflammatory response pathways, this observation supports the idea that RNase 7 possesses marked and promising modulatory effects on LPS‐induced innate immune activation.

### RNase 7 intracellular killing ability

An increasing number of recent studies have reported that intracellular bacteria can more readily acquire drug resistance and evade both immune system clearance and conventional antibiotic therapies [45, 46]. Infected host cells can protect intracellular bacteria from antimicrobial killing, facilitating their persistence and dissemination [47]. Moreover, most traditional antimicrobials have limited membrane permeability, which hinders their ability to kill intracellular bacteria effectively. Given these challenges, we aimed to explore whether RNase 7 could overcome these barriers by entering host cells and potentially promote intracellular bacterial clearance.

**Fig. 8:**
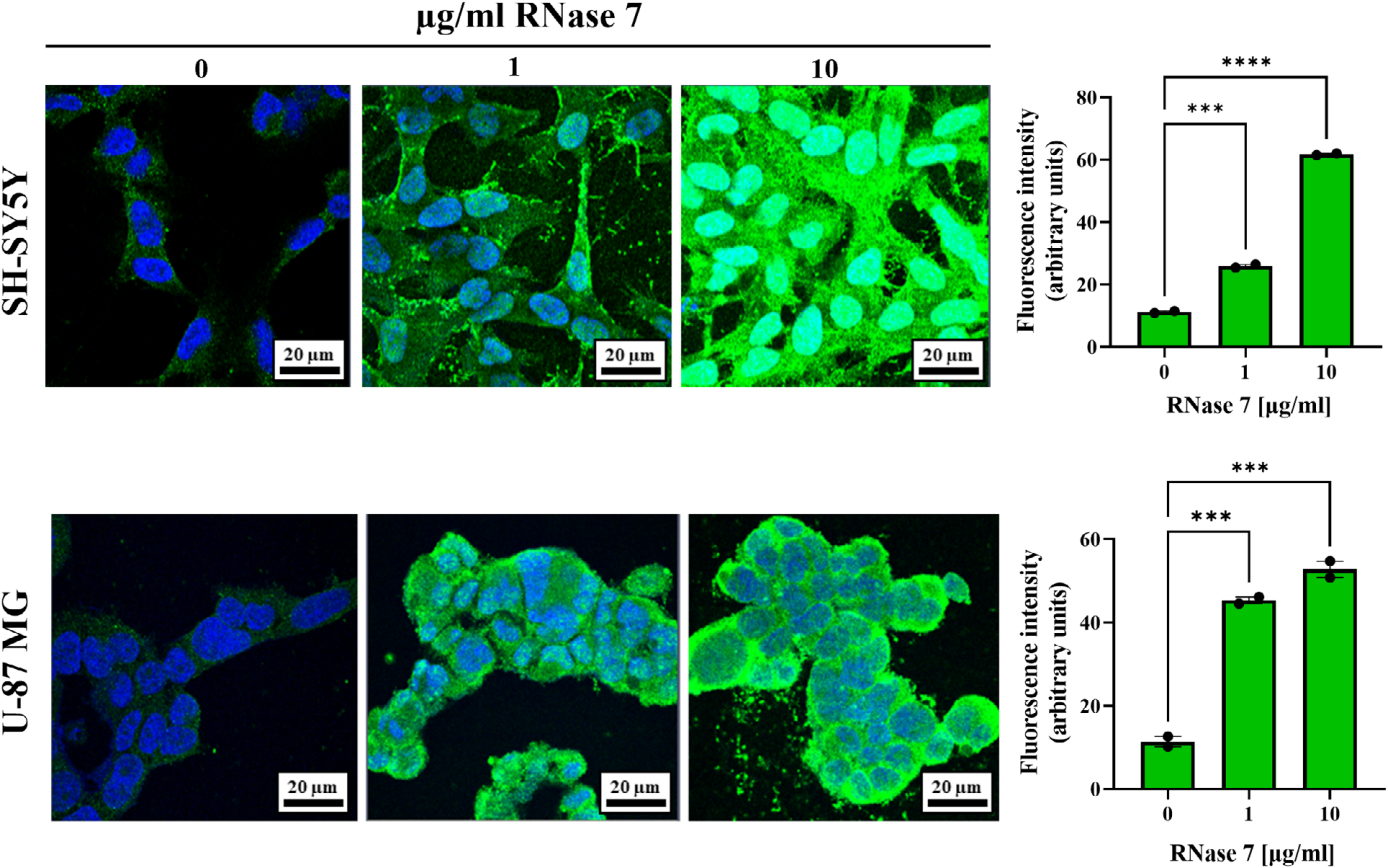
Analysis of RNase 7 internalization in SH‐SY5Y and U‐87 MG. A dose‐dependent increase in intracellular fluorescence signal associated with RNase 7 has been detected. Immunofluorescence analysis was performed on SH‐SY5Y and U‐87 MG cells treated for 1 h with two different doses of recombinant RNase 7 subsequently observed by Zeiss Confocal Laser scanning Microscope. LSM 700 at 63× magnification. Fluorescence intensity was measured by using imagej. The experiments were performed in monoplicate each on *n* = 2 biological replicates and reported as mean ± SEM. Statistical analyses were carried out by one‐way ANOVA, followed by Bonferroni's post‐test (***P < 0.001 or ****P < 0.0001). See Materials and methods section for experimental details.

**Fig. 9:**
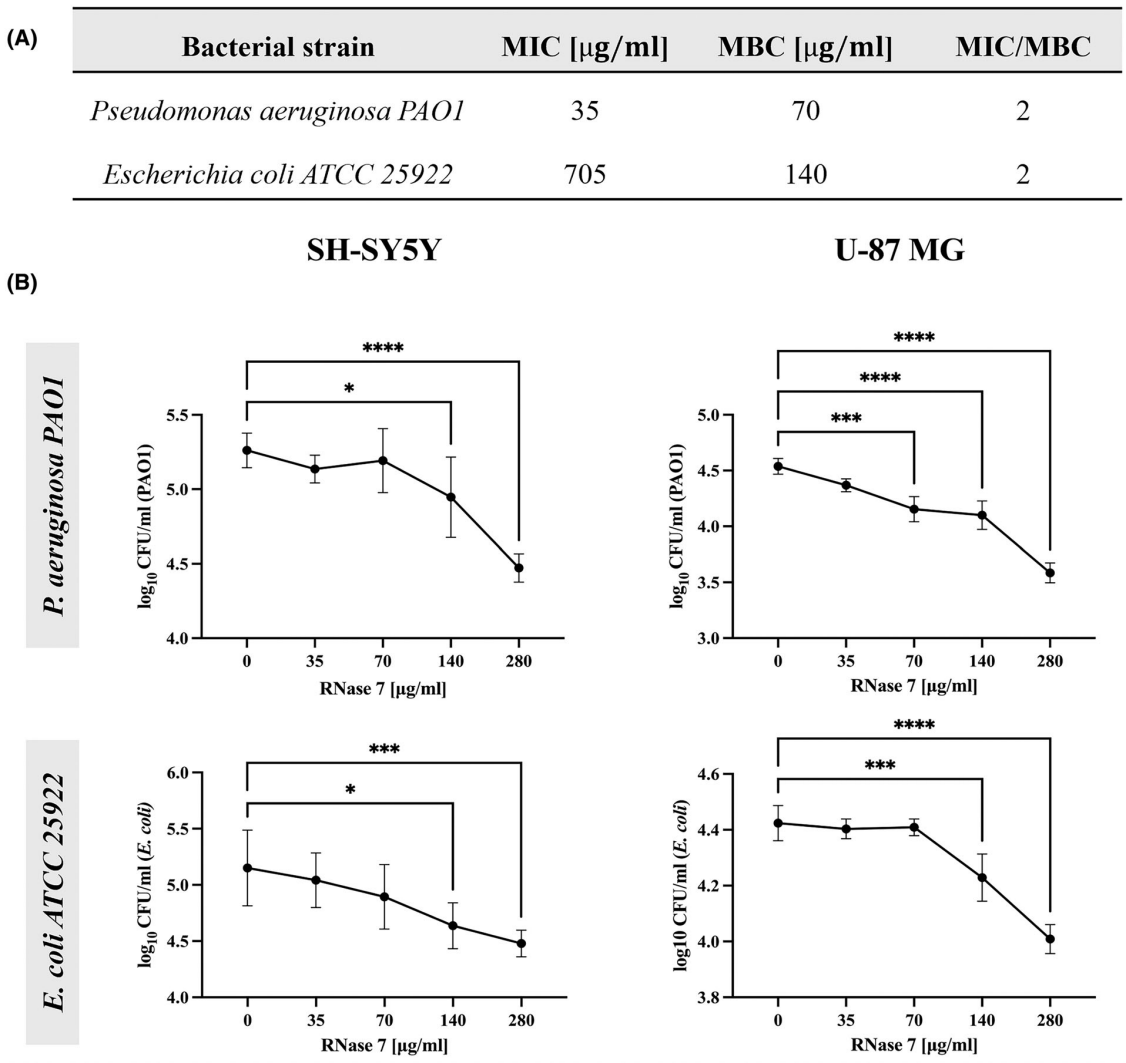
Intracellular clearance/killing induced by RNase 7 *Pseudomonas aeruginosa* O1 and *Escherichia coli* ATCC 25922 in SH‐SY5Y and U‐87 MG cells infected with *P. aeruginosa* O1 or *E. coli* ATCC 25922. (A) *In vitro* evaluation of antimicrobial activity of RNase 7 against *E. coli* ATCC 25922 and *P. aeruginosa* O1, used in (B). for the infection of both SH‐SY5Y and U‐87 MG cells. The experiments were performed in triplicate, each on *n* = 3 biological replicates, and reported as mean ± SEM. Statistical analyses were carried out by one‐way ANOVA, followed by Bonferroni's post‐test (*P < 0.05, ***P < 0.001 or ****P < 0.0001) versus unstimulated cells. See Materials and methods section for experimental details.

To address this, we first investigated the internalization of recombinant RNase 7 in SH‐SY5Y and U‐87 MG cells. Confocal Laser Scanning Microscopy (CLSM) confirmed efficient uptake of RNase 7 in both cell lines (Fig. 8), allowing us to design a preliminary experiment to assess its potential involvement in intracellular bacterial killing against *P. aeruginosa* 10 and *E. coli* O111:B4, both selected based on their pronounced susceptibility to RNase 7, as indicated by the MIC values reported in Fig. 9 (Panel A).

To test potential intracellular effects of RNase 7, cells were pretreated with it before infection, followed by washing, permeabilization, and plating of lysates for colony‐forming unit (CFU) counts (Materials and methods). As shown in Fig. 9 (Panel B), pretreated cells displayed a reduction in CFU counts when compared with untreated cells, and this suggests a potential intracellular contribution of RNase 7, although the proposed experimental setup does not allow to exclude with certainty a possible contribution of residual RNase 7 on the cell surface. Moreover, it is to note that concentrations of RNase 7 used in this experiment were relatively high, but they were also intentionally selected to verify its potential therapeutic applications in infection sites where higher local concentrations could be both achievable and relevant.

Nevertheless, despite these possible limitations, the observed trend seems encouraging and will certainly justify in‐depth investigations to clarify whether RNase 7 actively participates in intracellular killing mechanisms, such as autophagy or phagolysosomal pathways, in neuronal and glial contexts.

## Discussion

In the present study, we focused on examining functional properties of RNase 7 in human cells never explored to date in relation to this enzyme: SH‐SY5Y neuroblastoma and U‐87 MG glioma cells. The combined use of different approaches allowed us to detect that RNase 7 is constitutively expressed in both cell lines. Moreover, its expression is markedly influenced by different growth conditions, which could explain why, until now, only limited expression of this enzyme has been detected in the human brain according to “The Human Protein Atlas” database (https://www.proteinatlas.org/ENSG00000165799‐RNASE7/brain). These findings support the hypothesis that this protein may be part of a broader cellular stress response mechanism in the nervous system cells [25, 49].

Starting from this premise and considering the well‐recognized role of RNase 7 as a powerful paracrine agent, we investigated its potential host defense function using its recombinant form and testing SH‐SY5Y and U‐87 MG cells under specific non‐physiological conditions. With this approach, we explored the potential immunomodulatory and anti‐inflammatory effects of recombinant RNase 7 and its possible contribution to the intracellular control of pathogens, particularly in the context of neuroinflammation. Our data show that RNase 7 alone is neither toxic nor capable of inducing the release of pro‐inflammatory mediators; conversely, it significantly protects cells challenged with LPS. Specifically, we found that co‐administration of RNase 7 with LPS from different strains markedly attenuated the release of inflammatory mediators such as NO, TNF‐α, and IL‐6. In addition, RNase 7 reduced ROS accumulation following LPS stimulation as well as the extent of lipid peroxidation induced by ROS. Interestingly, RNase 7 exerted similar protective effects also on ROS release and lipid peroxidation in response to hydrogen peroxide, suggesting that in neurons this enzyme not only may act as a host defense agent and LPS scavenger, but it also exhibits complementary functional properties typically associated with other homologs, such as angiogenin [41] or RNase 4 [25, 49].

To further investigate a possible interaction between RNase 7 and LPS, we performed fluorescence anisotropy experiments, which allowed us to reveal a strong and specific binding of the enzyme to the endotoxin. These findings suggest that RNase 7 could directly target LPS molecules, possibly by preventing their recognition by receptors such as TLR4. In support of this hypothesis, we observed that co‐treatment of LPS‐stimulated SH‐SY5Y and U‐87 MG cells with RNase 7 markedly reduced *TLR4* gene expression, thus indicating a potential mechanism by which RNase 7 may attenuate inflammation through decreased TLR4 signaling. Moreover, internalization studies highlighted that recombinant RNase 7 can enter SH‐SY5Y and U‐87 MG cells, where it contributes to reducing the intracellular burden of phagocytosed bacteria.

These results, for the first time obtained in neuroblastoma and glioblastoma cells, are in line with what has been recently reported for RNase's ability to prevent intracellular *E. coli* infection in bladder epithelial cells [48] and protect against HSV infection in keratinocytes [49].

In addition, we found that RNase 7 can complex with LPS [23] and this supports a broader view of this protein as an antimicrobial effector with both extracellular and intracellular functions.

Similar dual functions of RNase 7 have also been reported in skin, where it limits RNA‐driven inflammation and its antimicrobial properties contribute to the control of *S. aureus* [25]. However, in inflammatory conditions such as atopic dermatitis, these protective functions are attenuated due to high levels of RNase inhibitor (RI), which blocks both the ribonuclease and antimicrobial activity of RNase 7. This highlights how the functional outcome of RNase 7 can be strongly shaped by the tissue microenvironment and suggests that analogous regulatory mechanisms may also influence its activity in the nervous system [29].

Overall, our results provide new insights into the role of RNase 7 in the nervous system, particularly under conditions of inflammation and oxidative stress. In this context, RNase 7 emerges as a potent modulator of the immune response, acting both as an LPS scavenger and as an antioxidant agent. Together with known studies in epithelial tissues, where RNase 7 controls RNA‐mediated inflammation and bacterial growth but is antagonized by RNase inhibitor during disease, our findings support a broader view of RNase 7 as a multifunctional effector whose activity is shaped by the tissue microenvironment. We believe that this study may open new avenues for exploring the therapeutic potential of RNase 7 in neuroinflammatory and neurodegenerative diseases, where inflammation and oxidative stress play a central role. Future work will be crucial to unravel the molecular mechanisms underlying these effects and to determine how regulatory factors, such as RI, may influence the balance between protective and pathogenic outcomes of RNase 7 activity in different tissues.

## Materials and methods

### Recombinant production of RNase 7

Recombinant RNase 7 was obtained by using an expression and purification protocol already described for other members of the RNase A Superfamily [41, 50, 51].

Briefly, the expression plasmid pET‐22b (+) encoding RNase 7 was used to transform competent *E. coli* strain BL21(DE3) (Thermo Fisher Scientific, Waltham, MA, USA). Cells were grown at 37 °C to an A_600nm_ = 0.6, then induced with 0.4 mm IPTG (Isopropyl β‐d‐1‐thiogalactopyranoside) and grown overnight. RNase 7 was expressed in inclusion bodies (IBs) obtained after sonication and centrifugation. IBs were then extensively washed to remove bacterial debris, solubilized and renatured under controlled redox conditions, and further purified by ultrafiltration and reverse‐phase HPLC [42, 50].

### Cell culture and treatments

The human neuroblastoma cell line SH‐SY5Y (RRID: CVCL_0019) and the human glioblastoma cell line U‐87 MG (RRID: CVCL_0022) were kindly provided by prof. Luisa Cigliano, Department of Biology, University of Naples Federico II. The cells were cultured in Dulbecco's modified Eagle medium (DMEM) supplemented with 10% fetal bovine serum (FBS), 1 mm L‐glutamine, 1% v/v penicillin/streptomycin solution (100 Unit·mL^−1^) and grown at 37 °C in 5% CO_2_.

Immunological activation was obtained by treating cells with 1 μg·mL^−1^ of LPS from *P. aeruginosa* 10 or *E. coli* O111:B4 (Merck KGaA, Darmstadt, Germany) for 24 h.

Oxidative stress was induced by incubating cells with 100 μm hydrogen peroxide (H_2_O_2_; Merck KGaA) for 24 h at 37 °C or with 500 μm sodium arsenite (SA) for 1 h.

Biocompatibility of recombinant RNase 7 at different doses was tested for 24 h as described below. For all other experiments, RNase 7 treatments were carried out by using two different concentrations (1 and 10 μg·mL^−1^).

To induce neuronal differentiation, SH‐SY5Y cells were incubated in a low‐serum medium (1.5% FBS) supplemented with 10 μm retinoic acid (RA; Sigma‐Aldrich®, St. Louis, MO, USA). Specifically, 3 × 10^5^ cells were plated in 6‐well plates for morphological evaluation and 6 × 10^5^ cells for RT‐qPCR analysis. Additionally, 2 × 10^4^ cells were plated in 96‐well plates for the assessment of anti‐inflammatory potential. The following day, the medium was replaced with fresh RA‐containing medium (1.5% FBS), which was changed every 2 days. Neuronal differentiation was monitored by bright‐field microscopy, which revealed progressive morphological changes of soma and neurite elongation with increased arborization, clearly detectable at day 3 and more pronounced at day 7 of differentiation (Fig. 3 Panel A). In parallel, neuronal maturation was evaluated by RT‐qPCR analysis of specific neuronal marker mRNAs (primer sequences in Table 1), which displayed a significant upregulation after 7 days of differentiation (Fig. 3 Panel B). After the differentiation period, the medium was removed, and the cells were subjected to the respective experimental treatments. All cell lines used in this study were routinely tested for Mycoplasma contamination using a PCR‐based assay and were confirmed to be Mycoplasma‐free throughout the duration of the experiments.

**Table 1:** Primer sequences of selected genes for SH‐SY5Y cells differentiation analysis.

Genes	Forward	Reverse
*NTRK2*	5′‐CGGGGACTTTGGGATGTC‐3′	5′‐CGCTTTCCGTCGTGAATTTC‐3′
*MAPT*	5′‐GCCACCAGGATTCCAGCAAA‐3′	5′‐TGGACTTGCTTAGTCGCAGA‐3′
*NRG1*	5′‐TGTGCGGAGAAGGAGAAAAC‐3′	5′‐GGCAGCGATCACCAGTAAAC‐3′
*SYP*	5′‐ACTATGGGCAGCAAGGCTAC‐3′	5′‐CTGGGCTTCACTGACCAGAC‐3′
*SHANK3*	5′‐CTGCCCTACCTGGAGTTTCG‐3′	5′‐TCAGGTTCGCCTTTGTGTGA‐3′

### Cell viability assays

Biocompatibility of RNase 7 on SH‐SY5Y and U‐87 MG cells was assessed by MTT method [52, 53]. Briefly, 2 × 10^4^ cells were first seeded in 96‐well plate for 24 h and then treated with increasing amounts of recombinant protein, LPS or combinations. Cells viability was analyzed at 570 nm by means of a Multi‐Mode Microplate Reader (Synergy™ H4, BioTek Instruments, Thermo Fisher Scientific, Winooski, VT, USA). Cell survival was expressed as the means of percentage values compared to untreated cells control.

### Antimicrobial activity

Antimicrobial activity of RNase 7 on *P. aeruginosa* O1 and *E. coli* ATCC 25922, was determined by using the broth microdilution method, as previously described [54]. Bacteria were grown to mid‐logarithmic phase in LB broth (Luria‐Bertani broth) at 37 °C, then diluted to 2 × 10^6^ Colony‐Forming Units (CFU)·mL^−1^ in Difco 0.5X Nutrient Broth (NB; Becton‐Dickenson, Franklin Lakes, NJ, USA) and mixed 1 : 1 v/v with two‐fold serial dilutions of antimicrobials. Following overnight incubation, MIC (Minimum Inhibitory Concentration) values were determined as the lowest protein concentration responsible for no visible bacterial growth. All the experiments were carried out on three independent replicates. To evaluate the bactericidal activity of RNase 7, the MBC (Minimum Bactericidal Concentration) was determined from the broth dilution of the MIC tests [55] by subculturing cell mixtures on LB agar plates. The MBC was defined as the lowest concentration of an antibacterial agent that killed ≥ 99.9% of bacterial cells.

### Endogenous RNase 7 analysis by enzyme‐linked immunosorbent assay (ELISA assay)

RNase 7 detection under different cell growth conditions was determined by ELISA. Briefly, 2 × 10^4^ cells were seeded in 96‐well plate and incubated overnight at 37 °C and 5% CO_2_. The next day, cells were then stimulated with different stressors (see Cell culture and treatments). Subsequently, a 96‐well plate was coated with 5 μg of total protein (both intracellular and secreted). The plate was incubated at 4 °C overnight, allowing the proteins to adhere to the bottom of the wells. After incubation, wells were rinsed with phosphate‐buffered saline (PBS) and then blocked with 5% bovine serum albumin (BSA) for 1 h at room temperature. Then the primary antibody mRNase 7 (Thermo Fisher Scientific, Waltham) was diluted in the blocking solution, added to each well, and incubated overnight at 4 °C.

After incubation, the wells were washed 3 times with PBS‐T (PBS 1X and 0.05% Tween‐20) before incubation with HRP‐conjugated secondary antibody, which was incubated for 1 h at room temperature.

At this point, the wells were rinsed 3 times in PBS‐T and for signal development, incubated in TMB for 10 min. The reaction was stopped with 0.5 m H_2_SO_4_ and then analyzed at 450 nm using a multi‐mode microplate reader (Synergy™ HTX, BioTek Instruments, Thermo Fisher Scientific). Calibration curves were obtained by the same procedure using recombinant RNase 7 to quantify the results.

### Western blot analyses

Proteins from SH‐SY5Y and U‐87 MG cells were quantified by Bradford method (Sigma‐Merck, Milan, Italy). A total of 30 μg of proteins were separated by SDS/PAGE, and then electro‐transferred to Immobilon‐PVDF membranes (Sigma‐Merck). To prevent non‐specific binding, membranes were blocked with 5% BSA and then probed overnight with the following primary antibodies: rabbit polyclonal anti‐PABP (Sigma‐Merck) and mouse monoclonal anti‐RNase 7 (Abcam, Cambridge, UK). After a washing step, the membranes were incubated with HRP‐conjugated goat anti‐mouse or anti‐rabbit IgG (1 : 5000) (ImmunoReagents Inc., Microtech SRL, Naples, Italy). Signals were developed using an ECL substrate and captured with a ChemiDoc Imaging System (Bio‐Rad Laboratories S.r.l., Milan, Italy). Densitometric evaluation of the bands was performed using imagej software (National Institutes of Health, Bethesda, MD, USA).

### Reverse transcription quantitative PCR (RT‐qPCR)

*RNase 7* gene expression was evaluated in SH‐SY5Y and U‐87 MG cells by RT‐qPCR. In brief, 3 × 10^5^ cells were plated and after 24 h, exposed to treatments (see Cell culture and treatments). Total RNA was then isolated using TRIzol™ Reagent (Thermo Fisher Scientific, Waltham) according to the manufacturer's instructions, and 1000 ng of RNA were used for cDNA synthesis using SuperScript™ IV VILO™ Master Mix (Thermo Fisher Scientific, Waltham) following the manufacturer's protocol. RT‐qPCR was performed by using SYBR GREEN PCR MASTER MIX (Thermo Fisher Scientific, Waltham) in the StepOnePlus™ Real‐Time PCR System (Thermo Fisher Scientific, Waltham). The expression of each gene detected (Table 2) was normalized to the transcript levels of *GAPDH* (housekeeping control) and reported as relative fold changes using the $2^{-\Delta \Delta C_{t}}$, or as $2^{-\Delta C_{t}}$ as appropriate, depending on the experimental objective (details are specified in the results).

**Table 2:** Primer sequences of selected genes.

Genes	Forward	Reverse
*RNase 7*	5′‐CCCTACACCCAGAGCATTCT‐3′	5′‐CCATCCCTCTAGAGCTCAGC‐3′
*TLR4*	5′‐TGGATACGTTTCCTTATAAG‐3′	5′‐GAAATGGAGGCACCCCTTC‐3′
*GAPDH*	5′‐CACCACACTGAATCTCCCCT‐3′	5′‐TGGTTGAGCACAGGGTACTT‐3′

### Immunofluorescence

Immunofluorescence analyses were performed on 2.5 × 10^4^ SH‐SY5Y or U‐87 MG cells seeded on glass coverslips in 24‐well plates and cultured for 24 h. After treatments (see Cell culture and treatments), cells were fixed in 4% paraformaldehyde (PFA), permeabilized in 0.1% Triton X‐100, and blocked with 1% BSA. Then cells were incubated overnight with primary antibodies, suitably diluted as indicated by the manufacturer, in 1% BSA. The primary antibody used was mouse Anti‐RNase 7 antibody [4C4] (Abcam Limited). After washing in PBS, the coverslips were incubated with secondary antibody Alexa488 conjugated goat anti‐mouse (Thermo Fisher Scientific, Waltham) for 1 h. Nuclei were stained with DAPI (Molecular Probes, Thermo Fisher Scientific, Waltham). After washing, coverslips were mounted in Mowiol® 4‐88. Images were acquired using Zeiss Confocal Laser scanning Microscope LSM 700 at 63× magnification. Fluorescence intensity analysis was then obtained by using imagej. Moreover, as control, to test whether cell‐associated RNase 7 is bound to the cell surface or internalized, we performed an acid wash experiment, following the details described in [56].

### DCFH‐Da assay

The ROS quantification assay was carried out by using the DCFH‐Da (2′,7′‐Dichlorofluorescin diacetate), following previously established protocols [57]. 2 × 10^4^ cells were seeded into 96‐well plate and incubated at 37 °C in a 5% CO_2_ atmosphere overnight. Then, cells were washed in PBS 1X, opportunely treated (see Cell culture and treatments), and incubated with 20 μm DCFH‐Da, at 37 °C for 40 min. The resulting fluorescence intensity was quantified using a Synergy™ H4 Multi‐Mode Microplate Reader (BioTek Instruments, Thermo Fisher Scientific) with excitation and emission wavelengths set at 485 and 532 nm, respectively.

### Lipid peroxidation analysis

Lipid peroxidation products [thiobarbituric acid reactive substances (TBARS) also known as malondialdehyde‐equivalents (MDA‐equivalents)] from SH‐SY5Y and U‐87 MG cells were measured by the thiobarbituric acid colorimetric assay, as previously reported [58, 59].

Briefly, cells were seeded in 6‐well plates at density of 2.5 × 10^5^ cells per well. Cells were then opportunely treated (see Cell culture and treatments), washed with PBS, counted, and centrifuged at 500 rpm for 5 min. After removal of supernatant, 0.5 mL of ice‐cold 40% trichloroacetic acid (TCA) and 0.5 mL of 0.67% of aqueous thiobarbituric acid were added to the pellet. The mixtures were heated at 90 °C for 15 min, then cooled in ice for 10 min, and centrifuged at 800 ***g*** for 10 min. The supernatant fractions were collected, and lipid peroxidation estimated spectrophotometrically at 530 nm. The amount of TBARS formed was calculated using a molar extinction coefficient of 1.56 × 10^5^·mol^−1^·cm^−1^ and expressed as nmol TBARS/10^6^ cells.

### Immunomodulatory activity analysis

The ability of RNase 7 to modulate the release of cytokines and the production of nitric oxide in SH‐SY5Y and U‐87 MG cells was measured via an ELISA (enzyme‐linked immunosorbent assay) and a Griess assay, respectively as reported in [60]. Cells (2 × 10^4^ cells per well) were seeded into 96‐well microtiter plates. The next day, the culture medium was discarded and replaced with a fresh medium containing either (a) a mixture of RNase 7 (1 or 10 μg·mL^−1^) and LPSs from *P. aeruginosa* 10 or *E. coli* O111:B4 (co‐incubation), (b) only RNase 7 (1 or 10 μg·mL^−1^) or (c) only LPSs from *P. aeruginosa* 10 or *E. coli* O111:B4. Cell supernatants were collected after 24 h of incubation at 37 °C and 5% CO_2_. The release of TNF‐α (tumor necrosis factor‐α) and IL‐6 (Interleukin‐6) was measured using specific ELISA kits (Thermo Fisher Scientific, Waltham), following the manufacturer instructions. The nitrite concentrations were determined via a colorimetric reaction using the Griess Reagent Kit for nitrite quantitation (Thermo Fisher Scientific, Waltham). Briefly, cell culture supernatants were mixed with equal volumes of N‐(1‐naphthyl) ethylenediamine (Component A) and sulfanilic acid (Component B) to form the Griess Reagent and incubated for 30 min at room temperature. The absorbance was measured at 548 nm using a 96‐well microplate reader (Synergy™ HTX, BioTek Instruments, Thermo Fisher Scientific, Winooski, VT, USA).

### Steady‐state fluorescence anisotropy

To determine the dissociation constant (*K*_d_) between RNase 7 and LPS from *E. coli* O111:B4 and LPS from *P. aeruginosa* 10, steady‐state fluorescence anisotropy experiments were performed. Briefly, a series of solution of RNase 7 (at fixed concentration of 1.5 μm) mixed with LPS *E. coli* O111:B4. (concentration range 0–4 μm) or LPS *P. aeruginosa* 10 (concentration range 0–3 μm) were prepared at the final volume of 1000 μL. The anisotropy of the protein was evaluated at the wavelength of 338 nm upon excitation at 280 nm by means of a FluoroMax‐4 spectrofluorometer (Horiba Scientific, Edison, NJ, USA) equipped with polarizers. The temperature was set at 25 °C and controlled with a Peltier system ensuring an accuracy of ±0.1 °C. The path length of the quartz cuvette was 1 cm. For the evaluation of the binding constants, a plot of Δ*r* = *r* – *r*_(0)_ (where *r* and *r*_(0)_ are the anisotropies of RNase 7 in the presence and in the absence of LPS, respectively) versus the total LPS concentration was drawn. Then, the experimental data were fitted with an equivalent and independent sites model Equation [61, 62] implemented in OriginLab software, allowing the evaluation of the dissociation constants and stoichiometry. All the experiment were performed in 10 mm sodium phosphate buffer, pH 7.4.

### Intracellular bacterial killing study

SH‐SY5Y and U‐87 MG cells (2 × 10*5* cells per well) previously seeded in 24‐well plate were treated with RNase 7 (up to 280 μg·mL^−1^) or medium alone (as control) for 24 h at 37 °C and 5% CO*2*. Cells were washed twice with PBS and subsequently infected with log‐phase *E. coli* ATCC 25299 or *P. aeruginosa* 10 at a multiplicity of infection (MOI) of 5 for 1 h at 37 °C and 5% CO₂. Extracellular bacteria were eliminated using a gentamicin protection assay (100 μg·mL^−1^ for 1 h), followed by three washes with PBS. Cells were then lysed with 0.1% Triton X‐100 for 5 min. Intracellular bacterial loads were determined by plating serial dilutions of the cell lysates onto agar plates. Colony‐forming units (CFUs) were recorded after 24 h to assess intracellular bacterial survival. [63].

### Statistical analyses

Statistical analyses were carried out by using graphpad prism (GraphPad Software, San Diego, CA, USA). Values are reported as the means ± SEM of biological replicates in at least three independent experiments (*P < 0.05, **P < 0.01, ***P < 0.001 or ****P < 0.0001) compared to the respective controls (one‐way ANOVA, followed by Bonferroni's post‐test) and ±SD for protein LPS binding analysis.

## Author contributions

RC contributed to the article in conceptualization, data curation, formal analysis, investigation, validation, and visualization. MC, IDN, IP, EP, and RO contributed in investigation, validation, and methodology. AB, AD, and EN contributed in formal analysis, supervision, and validation. EP was involved in conceptualization, funding acquisition, project administration, resources, and supervision. All authors were involved in editing the draft, and all agree to be accountable for all aspects of the work.

## Conflict of interest

The authors declare no conflict of interest.

## Data Availability

The data that support the findings of this study are available from the corresponding author [elipizzo@unina.it] upon reasonable request.
